# Spontaneous Pneumothorax After Complete Atrioventricular Block in a Patient With Chronic Obstructive Pulmonary Disease: A Case Report

**DOI:** 10.7759/cureus.116799

**Published:** 2026-09-23

**Authors:** Miho Yamanaka, Sakiko Honda, Emi Nishiki, Masatoshi Hori, Tatsuya Kawasaki

**Affiliations:** 1 Department of Cardiology, Matsushita Memorial Hospital, Moriguchi, JPN; 2 Department of Respiratory Medicine, Matsushita Memorial Hospital, Moriguchi, JPN; 3 Department of Emergency Medicine, Matsushita Memorial Hospital, Moriguchi, JPN

**Keywords:** atrioventricular block, bradycardia, chronic obstructive pulmonary disease, pneumothorax, respiratory pattern

## Abstract

Pneumothorax arises from a wide variety of causes, and chronic obstructive pulmonary disease (COPD) is the leading cause of secondary spontaneous pneumothorax.

We report an 84-year-old man with COPD who presented with breathing difficulty and was found to have a complete atrioventricular block with a ventricular rate of 41 beats per minute, together with a small spontaneous pneumothorax of the left apex. His symptoms had begun six days earlier with a self-reported heart rate of 40 beats per minute, and chest pain with worsening dyspnea developed three days thereafter. The pneumothorax was managed conservatively, and a permanent pacemaker was implanted after temporary pacing, with an uneventful clinical course. Given that cardiac dysfunction can alter the breathing pattern, the present case raises the possibility that the temporal sequence predisposed the emphysematous lung to pneumothorax.

## Introduction

Pneumothorax, defined as the presence of air within the pleural space, arises from a wide variety of causes and is generally classified as spontaneous, traumatic, or iatrogenic. Spontaneous pneumothorax is further divided into a primary form, which occurs in the absence of clinically apparent lung disease, and a secondary form, which develops as a complication of an underlying pulmonary disorder. Chronic obstructive pulmonary disease (COPD) is the most common cause of secondary spontaneous pneumothorax, in which rupture of subpleural blebs or emphysematous bullae allows air to enter the pleural cavity [1,2]. This secondary form tends to occur in older patients and carries a higher morbidity than the primary form [1,2]. Complete atrioventricular block, in turn, is a bradyarrhythmia in which atrial impulses fail to conduct to the ventricles, so that the ventricular rate is governed by a slower escape rhythm.

In this report, we present a patient with COPD who developed a spontaneous pneumothorax three days after the onset of complete atrioventricular block, and we discuss the possible link between the altered breathing associated with the bradyarrhythmia and the occurrence of pneumothorax.

## Case presentation

An 84-year-old man presented to the Emergency Department of our hospital because of breathing difficulty. He had been in his usual state of health until six days before presentation, when chest discomfort and breathing difficulty developed. The patient reported that the heart rate had been persistently around 40 beats per minute from that time until presentation on home blood pressure measurements, although it had usually been 60 to 80 beats per minute. His symptoms did not abate and, three days before presentation, chest pain suddenly developed. His breathing difficulty deteriorated, but the patient did not seek medical care until his scheduled visit to his physician. At the clinic, a diagnosis of complete atrioventricular block as well as pneumothorax on the left side was suspected, and the patient was transferred to our hospital. His medical history was notable for COPD, hypertension, gastroesophageal reflux disease, and osteoporosis. His medications included carbocisteine, fluticasone furoate/vilanterol, amlodipine, esomeprazole, and alendronate. He had an 80- to 120-pack-year smoking history, drank occasionally, did not use illicit drugs, and had no known allergies. His aunt had undergone pacemaker implantation.

On examination, he was alert but appeared ill. The blood pressure was 166/68 mmHg, the pulse rate was 40 beats per minute, the body temperature was 36.6°C, the respiratory rate was 24 breaths per minute, and the oxygen saturation was 98% while breathing oxygen supplement through a nasal cannula at a rate of 1 liter per minute. On chest auscultation, breath sounds were mildly diminished over the left hemithorax. Electrocardiography demonstrated right bundle branch block and complete atrioventricular block with a ventricular rate of 41 beats per minute (Figure 1A). Hyperinflated, hyperlucent lung fields consistent with underlying COPD, along with a small pneumothorax at the left apex, were confirmed on chest radiograph (Figure 1B, arrows) and computed tomography without administration of contrast material (Figure 1C, asterisks). Arterial blood gases had a pH of 7.441, partial pressure of carbon dioxide of 36.5 mmHg, partial pressure of oxygen of 95.4 mmHg, lactate of 6 mg/dL, and bicarbonate of 24.8 mmol/L. His complete blood cell count was normal except for hemoglobin of 13.0 g/dL, as were his electrolyte balance and renal and liver function tests. The high-sensitivity cardiac troponin T level was 0.016 ng/mL (reference value, <0.100), and the brain natriuretic peptide level was elevated at 245.5 pg/mL (reference value, ≤18.4). Bedside echocardiography showed normal chamber sizes and ventricular function without significant valvular heart disease or pericardial effusion.

**Figure 1:**
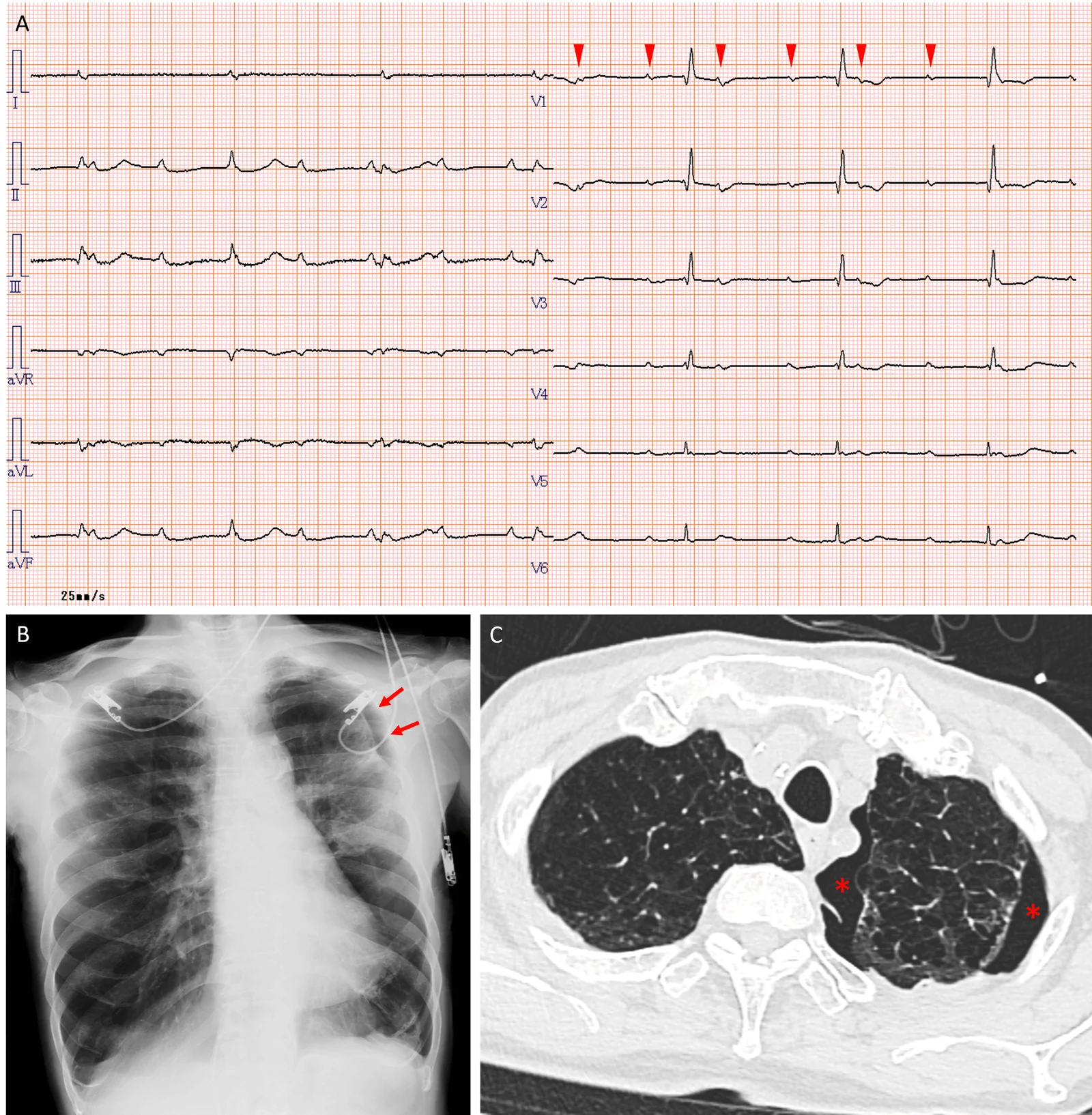
Electrocardiography and chest imaging Electrocardiography shows complete atrioventricular block with a ventricular response of 41 beats per minute and right bundle branch block (A). Arrowheads indicate P waves dissociated from the QRS complexes. Chest radiograph shows hyperinflated, hyperlucent lung fields along with a small pneumothorax at the left apex (B, arrows). Computed tomography of the chest without administration of contrast material confirms the presence of a pneumothorax at the left apex (C, asterisks).

A chest tube insertion for pneumothorax was waived because of its mild severity. A temporary pacing lead was inserted into the right ventricular apex through the right internal jugular vein. No improvement in the cardiac conduction system was observed during his hospital stay, and a permanent pacemaker was successfully implanted via the left subclavian vein under fluoroscopy and contrast venography to prevent deterioration of the left-sided pneumothorax. His clinical course was uneventful, with no progression of the left-sided pneumothorax. The patient was discharged in stable condition and was scheduled for follow-up at our hospital.

## Discussion

We described an 84-year-old man with underlying COPD who presented with a complete atrioventricular block and a small spontaneous pneumothorax of the left apex that had developed three days later. Chest imaging demonstrated hyperinflated, hyperlucent lung fields consistent with emphysema, providing an anatomical substrate for secondary spontaneous pneumothorax. The pneumothorax was managed conservatively without chest tube drainage owing to its mild severity, and a permanent pacemaker was implanted after temporary pacing, with an uneventful clinical course thereafter. The mildly elevated brain natriuretic peptide level, in the presence of normal ventricular function on echocardiography, was considered to reflect increased myocardial wall stress associated with the bradyarrhythmia rather than primary myocardial or valvular disease.

A variety of triggers have been proposed to precipitate spontaneous pneumothorax. Although the majority of episodes occur at rest rather than during vigorous physical exertion [3], sudden increases in transpulmonary pressure produced by coughing, sneezing, laughing, singing, or playing instruments can facilitate rupture of subpleural blebs or bullae. Furthermore, changes in ambient atmospheric pressure have also been implicated as a precipitating factor [4], which may in part account for the observed temporal clustering of cases. Common to these proposed mechanisms is a transient rise in the pressure gradient across the visceral pleura, which favors air leakage into the pleural space. However, in our patient, there was no episode possibly associated with the onset of spontaneous pneumothorax.

In the present patient, the pneumothorax became clinically apparent three days after the onset of complete atrioventricular block. The patient's ventricular rate was markedly reduced to approximately 40 beats per minute, and he had experienced worsening breathing difficulty during this period. Given that a change in breathing pattern accompanied the bradyarrhythmia, it is conceivable that the altered respiratory effort acted as a precipitating factor for the pneumothorax in a lung already rendered vulnerable by emphysematous change. In a patient with COPD, in whom subpleural bullae are fragile, even modest and repetitive increases in transpulmonary pressure arising from an unstable breathing pattern could plausibly promote bulla rupture [2].

In patients with cardiac dysfunction, ventilation is often augmented through a rapid and shallow breathing pattern rather than through an increase in tidal volume [5,6]. In the presence of expiratory flow limitation, such an increase in breathing frequency shortens the time available for expiration and may promote dynamic hyperinflation, with a resultant rise in end-expiratory alveolar pressure that could favor rupture of fragile subpleural bullae [7,8]. In the present patient, tachypnea with a respiratory rate of 24 breaths per minute was documented on admission, consistent with such a rapid breathing pattern. Although formal pulmonary function testing was not performed and any mechanistic explanation therefore remains speculative, it is conceivable that the increased respiratory frequency accompanying the complete atrioventricular block promoted dynamic hyperinflation and thereby predisposed the emphysematous lung to pneumothorax.

## Conclusions

We experienced a patient with COPD who developed a secondary spontaneous pneumothorax three days after the onset of complete atrioventricular block. Although a definitive causal relationship cannot be established from a single observation, the temporal sequence and the tachypnea accompanying the bradyarrhythmia raise the possibility that an altered breathing pattern served as a trigger for pneumothorax in an emphysematous lung. Clinicians should be aware that cardiac dysfunction, including bradyarrhythmia, tachyarrhythmia, and heart failure, may be associated with respiratory complications, and careful attention to respiratory status is warranted in such patients, particularly when an underlying pulmonary disease is present.
